# 2D Ultrathin Iron Doped Bismuth Oxychloride Nanosheets with Rich Oxygen Vacancies for Enhanced Sonodynamic Therapy

**DOI:** 10.1002/adhm.202301497

**Published:** 2023-06-15

**Authors:** Miaomiao Wu, Jiaxi Yong, Huayue Zhang, Zhiliang Wang, Zhi Ping Xu, Run Zhang

**Affiliations:** ^1^ Australian Institute for Bioengineering and Nanotechnology (AIBN) The University of Queensland St Lucia QLD 4072 Australia; ^2^ Institute of Biomedical Health Technology and Engineering and Institute of Systems and Physical Biology Shenzhen Bay Laboratory Shenzhen 518107 P. R. China

**Keywords:** 2D ultrathin nanosheets, bismuth oxychloride, cancer treatment, inorganic sonosensitizers, sonodynamic therapy

## Abstract

Sonodynamic therapy (SDT) combines ultrasound and sonosensitizers to produce toxic reactive oxygen species (ROS) for cancer cell killing. Due to the high penetration depth of ultrasound (US), SDT breaks the depth penetration barrier of conventional photodynamic therapy for the treatment of deeply seated tumors. A key point to enhance the therapeutic efficiency of SDT is the development of novel sonosensitizers with promoted ability for ROS production. Herein, ultrathin Fe‐doped bismuth oxychloride nanosheets with rich oxygen vacancies and bovine serum albumin coating on surface are designed as piezoelectric sonosensitizers (BOC‐Fe NSs) for enhanced SDT. The oxygen vacancies of BOC‐Fe NSs provide electron trapping sites to promote the separation of e^−^‐h^+^ from the band structure, which facilitates the ROS production under the ultrasonic waves. The piezoelectric BOC‐Fe NSs create a built‐in field and the bending bands, further accelerating the ROS generation with US irradiation. Furthermore, BOC‐Fe NSs can induce ROS generation by a Fenton reaction catalyzed by Fe ion with endogenous H_2_O_2_ in tumor tissues for chemodynamic therapy. The as‐prepared BOC‐Fe NSs efficiently inhibited breast cancer cell growth in both in vitro and in vivo tests. The successfully development of BOC‐Fe NSs provides a new nano‐sonosensitiser option for enhanced SDT for cancer therapy.

## Graphical abstract

Herein, it is reported that the development of monodispersed iron doped bismuth oxychloride nanosheets with uniform morphology and size as a novel sonosensitizer for enhanced sonodynamic therapy. The nanosheets are featured with rich oxygen vacancies and high piezoelectric properties that promote the reactive oxygen species generation under ultrasound irradiation for deeply seated cancer cells killing both in vitro and in vivo.

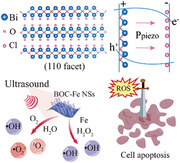

## 1 Introduction

Sonodynamic therapy (SDT) applies ultrasound (US) to activate sonosensitisers to produce reactive oxygen specials (ROS) for tumor cell apoptosis. SDT has emerged as a non‐invasive and promising therapeutic strategy for cancer treatment.^[^ ^1^ ^]^ As an alternative to conventional photodynamic therapy (PDT) that has limited tissue penetration depth (<1 cm), SDT can reach deeper tissue (more than 7 cm) to kill the deeply seated tumor cells with minimal damage to nearby healthy tissues.^[^ ^2^ ^]^ Sonosensitiser is the key component for SDT, in which the deep penetration of ultrasound activates the sonosensitiser to produce a large amount of ROS for the treatment.^[^ ^3^ ^]^ There are two categories of sonosensitisers, including organic molecules and inorganic nanoparticles.^[^ ^4^ ^]^ Traditional organic sonosensitisers are porphyrin‐based compounds, including hematoporphyrin monomethyl ether (HMME), photoporphrin IX (PpIX), a gallium–porphyr (ATX‐70) and their derivatives,^[^ ^5^ ^]^ while the application of these organic sonosensitisers are hampered due to their low bioavailability, poor stability and limited tumor enrichment.^[^ ^6^ ^]^ Inorganic semiconductor sonosensitisers, such as titanium dioxide (TiO_2_),^[^ ^7^ ^]^ black phosphorus (BP),^[^ ^8^ ^]^ and bismuth‐based nanomaterials^[^ ^9^ ^]^ are recently developed as new therapeutic agents for enhancing the efficiency of SDT. These sonosensitisers have their unique energy level structure, good chemical and physical stability.^[^ ^10^ ^]^ However, SDT performance of these inorganic semiconductors is limited due to the low quantum yields of ROS generation. To tackle this problem, defect engineering is adopted to the development of semiconductor sonosensitisers for enhanced SDT. It has been reported that doping metal ions in the nanostructure could reduce the electron (e^−^)‐hole (h^+^) recombination and facilitate trapping the excited electrons.^[^ ^11^ ^]^ Therefore, the sonodynamic effect could be augmented after introduction of metal ions.^[^ ^12^ ^]^ Nevertheless, the development of high efficiency inorganic semiconductor sonosensitisers with long‐term stability and good tumor accumulation is still in its infancy.

The theory of cavitation effect is now accepted as the main mechanism of SDT,^[^ ^13^ ^]^ although the exact process of SDT is still ambiguous.^[^ ^14^ ^]^ The cavitation effect is a dynamic process in which the cavitation nucleus in liquid undergoes shock, expansion, contraction, and implosion under certain US irradiation.^[^ ^15^ ^]^ When the cavitation bubbles rapidly collapse, the intertrial cavitation will further induce extreme conditions, including high temperature, pressure, and sonoluminescence.^[^ ^16^ ^]^ These conditions may provide adequate stimulation to activate sonosensitisers for ROS generation. Taking advantages of the high‐pressure amplitudes (almost 1 × 10^8^ Pa)^[^ ^17^ ^]^ caused by the bubble collapse, piezoelectric materials have emerged as a new kind of sonosensitisers,^[^ ^14^, ^18^ ^]^ such as layered bismuth‐based nanosheets,^[^ ^19^ ^]^ perovskite,^[^ ^20^ ^]^ and wurtzite crystal structure materials like zinc oxide (ZnO)^[^ ^21^ ^]^ and gallium nitride (GaN).^[^ ^22^ ^]^ Under the mechanical stress induced by US irradiation, the structure of piezoelectric crystals is compressed, twisted, and/or pulled, resulting in a variation of the surface charge density and a build‐in electric field in the materials. The separated e^−^‐h^+^ pairs are driven to the opposite surfaces while the recombination of charge carriers is suppressed by the piezotronics effect, thereby improving ROS production via the reaction with surrounding molecular H_2_O and O_2_.^[^ ^23^ ^]^

**Scheme 1:**
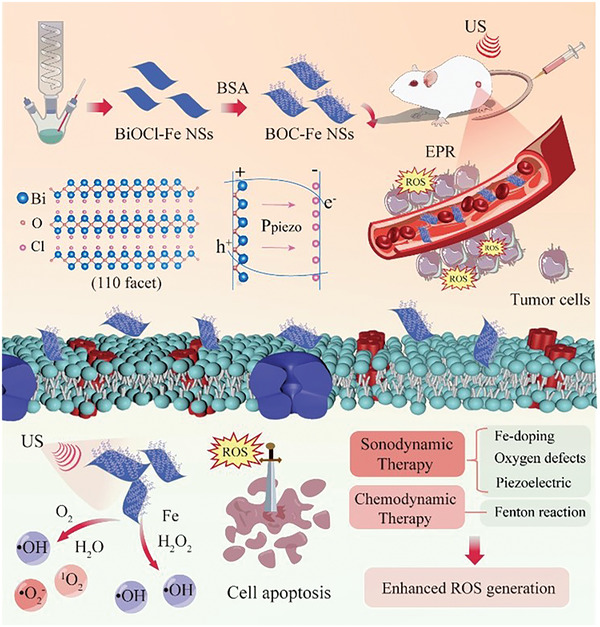
Schematic illustration the development of iron‐doped BiOCl NSs coated with BSA, BiOCl‐Fe NSs@BSA (BOC‐Fe NSs) as the sonosensitiser for enhanced tumor SDT.

As a layered semiconductor, bismuth oxychloride (BiOCl), consisting of a [Bi_2_O_2_]^2+^ layer sandwiched between two slabs of halogen ions, has been investigated frequently as a photocatalyst.^[^ ^24^ ^]^ Ultrathin 2D BiOCl nanosheets (NSs) are featured with high piezoelectric properties, allowing for their photo‐/piezo‐catalysis applications.^[^ ^25^ ^]^ Nevertheless, the piezoelectric properties of BiOCl NSs with metal dopants and defects, and particularly their biomedical application, have not been investigated yet. Herein, ultrathin iron‐doped BiOCl NSs (BiOCl‐Fe NSs) with oxygen defects were developed as a new piezoelectric sonosensitiser for the enhanced SDT in this research (**Scheme** **1**). By a robust one‐pot thermal decomposition approach, BiOCl‐Fe NSs with uniform morphology and size were synthesized. Coating with bovine serum albumin (BSA) provided BiOCl‐Fe@BSA NSs (BOC‐Fe NSs) with excellent biocompatibility and colloidal stability. Oxygen defects in the BOC‐Fe NSs structure could provide extra electron trapping sites to further promote the separation of e^−^‐h^+^ from the band structure. As a result, these BOC‐Fe NSs exhibited higher sonodynamic ROS production efficiency than that of pure BiOCl@BSA NSs (BOC NSs) under mild US irradiation. The piezoelectric characteristic of BOC‐Fe NSs enabled the formation of a built‐in piezopotential field under high pressure from the US‐mediated bubble collapse, accelerating the ROS generation. Additionally, BOC‐Fe NSs induced ROS generation by Fenton reaction catalyzed by Fe ion,^[^ ^26^ ^]^ to further enhance the cytotoxicity. We found that as‐prepared BOC‐Fe NSs efficiently inhibited 4T1 breast cancer cell growth in both in vivo and in vitro tests. Therefore, this research successfully developed a new sonosensitiser based on ultrathin defect‐rich BOC‐Fe NSs for highly efficient breast cancer cell killing, advancing the cancer SDT using 2D ultrathin nanosheets with strong piezotronic effects.

## 2 Results and Discussion

**Figure 1:**
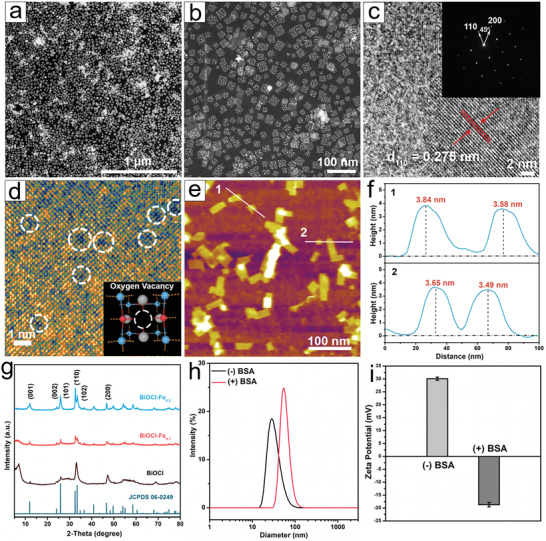
Characterization of the prepared BiOCl‐Fe_0.1_ NSs. HAADF‐STEM images in dark field a) in a large scale and b) in a small size. c) HRTEM image (inset: SAED image). d) Magnified HRTEM image with simulated oxygen vacancy model image (inset). e,f) AFM image and the corresponding height profiles. g) XRD pattern of BiOCl, BiOCl‐Fe_0.1_, and BiOCl‐Fe_0.2_ NSs. h) DLS size distribution and i) Zeta potential of BiOCl‐Fe_0.1_ without and with BSA coating.

Monodispersed BiOCl‐Fe NSs with uniform morphology and size were synthesized following a robust thermal decomposition approach. The high‐angle annular dark‐field scanning transmission electron microscopy (HAADF‐STEM) image shows that BiOCl‐Fe_0.1_ NSs (mole ratio of Fe/Bi was 0.1) feature a square morphology with an average size of 18.05 ± 1.5 nm (width) × 21.16 ± 0.7 nm (length) (**Figure** **1**a,b; Figure S1, Supporting Information). High‐resolution TEM (HRTEM) image (Figure 1c) and selected area electron diffraction (SAED) image (inset of Figure 1c) reveal their crystalline characteristics, in which the dominant lattice was the (110) plane of BiOCl crystal with the lattice space of ≈0.275 nm. SAED pattern shows that the angle between adjacent spots was 45 degrees, being identical to the theoretical value between the (110) and (200) planes of tetragonal BiOCl (Figure 1c inset). Through analyzing false color image of HRTEM (Figure 1d), the absence of atoms was identified (white cycles), suggesting the existence of oxygen vacancies (O_V_) on the (110) surface of BiOCl‐Fe_0.1_ NSs (Figure 1d inset). The presence of O_V_ was also confirmed by electron paramagnetic resonance (EPR) analysis (Figure S2, Supporting Information). EPR signal at g = 2.002 was observed, which could be assigned the oxygen vacancy defects.

The thickness of BiOCl‐Fe_0.1_ NSs was estimated to be ∼3.64 nm via atomic force microscopy (AFM) analysis (Figure 1e,f), which is consistent with the height of five‐unit cells of [Cl—Bi—O—Bi—Cl] along the (001) axis.^[^ ^25a^ ^]^ In addition, elemental mapping images of BiOCl‐Fe_0.1_ NSs show the uniform distribution of Bi, O, Cl, and Fe in the structure (Figure S3, Supporting Information). The successful doping of Fe in BiOCl NSs was further confirmed by the energy dispersive X‐ray spectroscopy (EDS) (Figure S4, Supporting Information), in which the emission peaks of Bi, O, Cl, Fe were clearly identified. To investigate the ROS generation performance of BiOCl‐Fe NSs under US, different mole ratios of Fe/Bi in BiOCl‐Fe NSs (0.05, 0.1, 0.2, and 0.4) and pure BiOCl NSs were synthesized using the same thermal decomposition method. The BiOCl‐Fe NSs with different Fe/Bi element ratios and the pure BiOCl NSs showed similar morphology, indicating that the Fe doping did not affect the crystal structure of nanosheets (Figure S5a–e, Supporting Information). Moreover, the crystal structure of as‐synthesized BiOCl‐Fe_0.1_ and BiOCl‐Fe_0.2_ NSs was well matched with the pure BiOCl pattern (JCPDS: 06–0249) (Figure 1g).

The surface of as‐prepared BiOCl‐Fe NSs was then modified with bovine serum albumin (BSA) coating to yield BiOCl‐Fe@BSA NSs (BOC‐Fe NSs) with elevated biocompatibility and colloidal stability. As illustrated in Figure 1h, dynamic light scattering (DLS) measurement of BOC‐Fe NSs showed an increase in the average hydrodynamic particle size from 35 to 41 nm after coating with BSA (Figure S6a, Supporting Information). The TEM image confirms the good dispersion of BOC‐Fe NSs in PBS buffer (Figure S6b, Supporting Information). The changes of the zeta potential from 30.1 to −20.2 mV further confirmed the successful BSA coating on the BOC‐Fe nanosheet surfaces (Figure 1i). Fourier transform infrared (FTIR) spectra were collected to determine the surface compositions of BSA, bare BiOCl‐Fe NSs, and BOC‐Fe NSs (Figure S7, Supporting Information). The characteristic peaks from both BSA and BiOCl‐Fe were identified in the pattern of BOC‐Fe NSs. The characteristic peaks at 1635 and 1513 cm^−1^ are attributed to amide groups of BSA, at 2920 and 2850 cm^−1^ assigned to ‐C‐H band stretching vibration from the surfactants of BiOCl preparation, and that at 964 and 721 cm^−1^ to M—O (M = Bi, Fe) vibrations.

**Figure 2:**
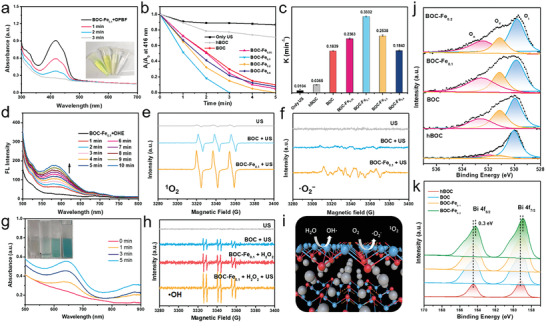
SDT and CDT performance of BOC‐Fe NSs. a) SDT performance of BOC‐Fe_0.1_ NSs using the DPBF probe. b,c) Comparison of SDT effects of BOC‐Fe_0.05_, BOC‐Fe_0.1_, BOC‐Fe_0.2_, BOC‐Fe_0.4_, BOC, and hBOC. d) Using the DHE probe for testing superoxide (·O_2_^−^) generation. e,f) ERP analysis of ^1^O_2_ and ·O_2_^−^ generation from US irradiated BOC‐Fe_0.1_ NSs by TEMP and DMPO. g) CDT performance of BOC‐Fe_0.1_ NSs using TMB as a probe and h) ERP analysis of ·OH generation from US irradiated BOC‐Fe_0.1_ NSs by PBN. i) The proposed reaction of SDT and CDT. j) O 1s and k) Bi 4f peaks in XPS spectra of BOC‐Fe_0.1_, BOC‐Fe_0.2_, BOC, and hBOC.

Next, the ROS generation performance of BOC‐Fe NSs, including SDT and chemodynamic effects, was investigated by detection of ROS using responsive probes. After US irradiation (50 kHz; 1 W cm^−2^; 3 min), 1,3‐diphenylisobenzofuran (DPBF) was employed as the probe for detection of singlet oxygen (^1^O_2_). As shown in **Figure** **2**a, the characteristic absorption of DPBF at 416 nm significantly decreased within 3 min of US irradiation, suggesting the substantial production of ^1^O_2_. For comparison, hydrothermal BiOCl NSs (hBOC) with the similar size and morphology (Figure S8a, Supporting Information) but without defects were prepared following the previous method.^[^ ^25a^ ^]^ The ROS generation rate of BOC‐Fe NSs with various mole ratios (BOC‐Fe_0.05_, BOC‐Fe_0.1_, BOC‐Fe_0.2_, BOC‐Fe_0.4_) were compared with BOC NSs and hBOC. Under US irradiation, all BOC‐Fe NSs and BOC NSs quickly induced higher amount of ROS than that of hBOC (Figure 2b; Figure S8b, Supporting Information), indicating that oxygen defects and doping Fe indeed improved the sonodynamic effect of the ultrathin BOC NSs. With the increase of Fe doping amount (0, 0.05, 0.1, 0.2, and 0.4), the ROS generation efficiency of BOC‐Fe NSs increased initially and then decreased (Figure 2c). The BOC‐Fe_0.1_ showed the higher sonodynamic performance than the other BOC‐Fe NSs (Figure 2b,c). Moreover, the ROS generation efficiency of BOC‐Fe_0.1_ was not changed after 4 times US irradiation within 15 days (Figure S9, Supporting Information), which is in sharp contrast to the rapid reduction in the efficiency of Rose Bengal (RB) (Figure S10, Supporting Information). The above data indicate that the highly stable BOC‐Fe_0.1_ NS can sustainably generate ROS for enhanced SDT. The ROS generation rate of BOC‐Fe NSs (k = 0.333) was 5− and 3− times higher than of that of TiO_2_ (k = 0.057) and BaTiO_3_ (k = 0.095) under the same US irradiation setting (Figure S11, Supporting Information), demonstrating the superior sonodynamic efficiency of BOC NSs. As a result, BOC‐Fe_0.1_ was chosen as the optimal sonosensitiser for the following SDT experiments.

Similarly, fluorescent probe dihydroethidium (DHE) was used to measure the generation of superoxide (·O_2_^−^) upon US irradiation of BOC‐Fe_0.1_ NSs (Figure 2d). The fluorescence intensity of DHE was gradually increased upon US irradiation, indicating the sustained generation of ·O_2_^−^. In addition, EPR analysis was employed for further confirm the generation of ^1^O_2_ and ·O_2_^−^, where ^1^O_2_ was trapped by 2,2,6,6‐tetra‐methylpiperidine (TEMP) (Figure 2e) and ·O_2_^−^ by 5,5‐dimethyl‐pyrroline N‐oxide (DMPO) (Figure 2f). The group of BOC‐Fe_0.1_ NSs + US showed the stronger intensity in both ^1^O_2_ and ·O_2_^−^ analyses compared with control groups of US only and BOC NSs + US, indicating the higher amount of ^1^O_2_ and ·O_2_^−^ generation during US irradiation.

The production of hydroxyl radicals (·OH) was then determined by using 3,3,5,5‐tetramethylbenzidine (TMB) as the probe. In the presence of ·OH, the oxidation of TMB leads to the emerging of characteristic absorption peak at 662 nm (Figure 2g). With the addition of H_2_O_2_ (50 µM), the absorption peak at 662 nm gradually increased. The solution color changed from colorless to blue, and then dark blue (inset digital photos), indicating the formation of ·OH through the Fenton reaction. More interestingly, XPS analysis of Fe ion in BOC‐Fe_0.1_ NSs demonstrated the presence of Fe^3+^/Fe^2+^ (Figure S12a–c, Supporting Information). The Fe^3+^/Fe^2+^ pair existed in the BOC‐Fe_0.1_ NSs could react with hydrogen peroxide (H_2_O_2_) to produce hydroxyl radicals (·OH) via Fenton reactions.^[^ ^27^ ^]^ In EPR analysis where the ·OH was trapped by N‐tert‐Butyl‐*α*‐phenylnitrone (PBN), the EPR signal of BOC‐Fe_0.1_ NSs with H_2_O_2_ was obtained, corroborating the generation of ·OH through Fenton reactions for chemodynamic therapy (CDT). Moreover, EPR signal of BOC‐Fe_0.1_ NSs + H_2_O_2_ with US irradiation was almost doubled in comparison with that without US irradiation (Figure 2h). These observations confirmed that BOC‐Fe_0.1_ NSs is an excellent material for ·OH production through US irradiation and Fenton reactions. As illustrated in Figure 2i, the electrons from the surface of BOC‐Fe_0.1_ NSs may react with O_2_ to generate ^1^O_2_ and ·O_2_^−^, whereas the holes may combine with H_2_O to produce ·OH.

Upon US irradiation, the enhanced ROS generation of BOC‐Fe_0.1_ NSs is ascribed to the Fe doping and oxygen defects. To evaluate the oxygen vacancy of Fe‐doped BOC NSs, X‐ray photoelectron spectroscopy (XPS) analysis of elemental O and Bi was conducted (Figure 2j,k). The oxygen vacancy in the lattice leads to the change of electron density in the surrounding oxygen atoms, which can be indicated by shift of oxygen's binding energy in XPS analysis.^[^ ^28^ ^]^ As shown in Figure 2j, three typical peaks at ≈530.1, 531.1, and 532.4 eV (labeled as O_L_, O_V_, O_A_) were attributed to lattice oxygen, oxygen vacancy, and surface absorbed oxygen species, respectively. In comparison with hBOC, substantial increase of the oxygen defect amount of BOC NSs and BOC‐Fe NSs was observed. The specific ratio of O_V_ in these nanosheets was 9% (hBOC), 30% (BOC), 24% (BOC‐Fe_0.1_), and 19% (BOC‐Fe_0.2_) (Table S1, Supporting Information), respectively. In the XPS pattern of Bi (Figure 2k), the binding energy of Bi 4f in BOC NSs and BOC‐Fe NSs shifted to lower energy (0.3 eV shift) compared with hBOC, indicating the decrease in the coordination number of Bi^3+^. No significant shift of Cl 2p's binding energy was observed (Figure S12d, Supporting Information), implying that the oxygen defects were in the (Bi_2_O_2_)^2+^ layer. The abundance of oxygen vacancies in BOC NSs altered the electron structure in the lattice and promoted quick charge transfer during the US irradiation, and thus enhanced the ROS generation.^[^ ^28^ ^]^ Excesses oxygen vacancies in BOC NSs (30%) may accelerate the interfacial recombination of e^−^ and h^+^, which in turn inhibits the ROS production.^[^ ^29^ ^]^

**Figure 3:**
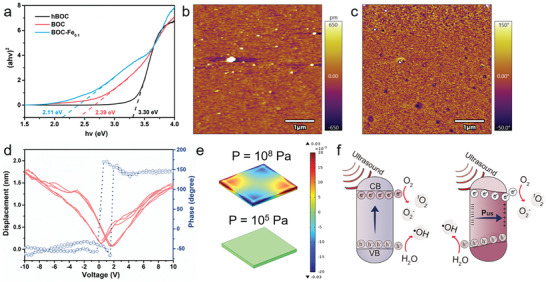
The proposed piezoelectric mechanism for enhanced sonodynamic efficiency. a) Bandgaps of BOC, and BOC‐hydrothermal, and BOC‐Fe_0.1_. b) Topographic image, c) phase image, and d) phase hysteresis (blue) and butterfly (red) loop of BOC‐Fe_0.1_. e) COMSOL simulation of the piezoelectric potential distribution in BOC nanosheets under cavitation pressure (P = 10^8^ Pa) and acoustic pressure (P = 10^5^ Pa). f) Simulating response of non‐piezoelectric sensitizer versus piezoelectric sensitizer under US irradiation.

To further explore the underlying mechanism of the highest sonodynamic efficiency of BOC‐Fe_0.1_ NSs, the solid ultraviolet spectra of hBOC, BOC NSs, and BOC‐Fe_0.1_ NSs were measured (Figure S13, Supporting Information) and the band gaps (Eg) of these nanosheets were calculated. As shown in **Figure** **3**a, the bandgap of BOC‐Fe_0.1_ NSs (2.11 eV) was significantly narrowed than that of hBOC (3.3 eV) and BOC NSs (2.39 eV). It has been reported that the level of band gaps plays an important role in ROS generation.^[^ ^12^, ^30^ ^]^ The narrowed band gap of BOC‐Fe_0.1_ NSs is beneficial for enhancing electron‐hole separation efficiency under US irradiation.

Subsequently, the piezoelectric property of BOC‐Fe_0.1_ NSs was confirmed by piezoresponse force microscopy (PFM) technique. The topographic image, corresponding phase image and amplitude image are shown in Figure 3b,c; Figure S14 (Supporting Information), respectively. The phase image shows that a clear phase difference and the presence of 150° domains demonstrated the piezoelectric activity of BOC‐Fe_0.1_ NSs (Figure 3c). The hysteresis amplitude‐voltage curve and butterfly loop of BOC‐Fe_0.1_ NSs were observed when the reverse voltage was changed from 10 to −10 V (Figure 3d). The phase inversion was about 150° in the localised piezoelectric hysteresis curve. These PFM data clearly demonstrate the piezoelectricity of BOC‐Fe_0.1_ NSs.

In addition, COMSOL multiphysics software was used to simulate the distribution of nanosheets’ piezoelectric potential. When US propagates through a medium, the acoustic pressure (10^5^ Pa)^[^ ^31^ ^]^ is generated while cavitation effect produces a shockwave with a pressure of ≈10^8^ Pa on the sonosensitiser.^[^ ^32^ ^]^ Both mechanical deformation and piezoelectric effect can take effect on the nanosheets. A typical simulation for BOC nanosheets with width = 20 nm, length = 20 nm, thickness = 5 nm, and Pressure (P) = 10^8^ Pa/10^5^ Pa is demonstrated in Figure 3e. The induced potential difference was 40 mV at the pressure of 10^8^ Pa, while negligible difference of potential was obtained at the pressure of 10^5^ Pa. As illustrated in Figure 3f, under ultrasound stimulation, the high pressure generated could activate the piezoelectric material to enhance the sonodynamic effect and amplify ROS generation. Together with the oxygen vacancy of BOC‐Fe_0.1_ NSs, piezoelectric activity may further promote the ROS production for enhanced SDT for cancers in vitro and in vivo.

**Figure 4:**
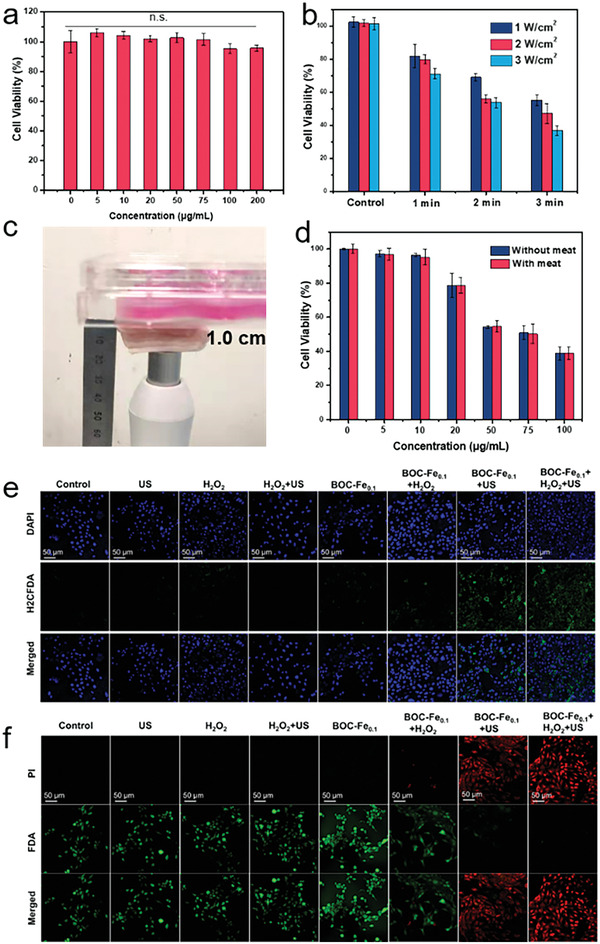
In vitro assessments of BOC‐Fe_0.1_ NSs sonosensitiser‐mediated SDT. a) MTT cell viability assay of BOC‐Fe_0.1_ NSs with different concentrations. b) Relative viability of BOC‐Fe_0.1_ NSs incubated 4T1 cells at different US power and irradiation period. c) Image of in vitro simulated deep tissue model of SDT. d) Relative viability of BOC‐Fe_0.1_ NSs incubated 4T1 cells after SDT with and without meat (≈1 cm). e) Confocal images of 4T1 cells treated with H_2_DCF‐DA after different conditions. f) Confocal images of FDA/PI stained with 4T1 cells after various treatments.

We next evaluated the SDT performance of BOC‐Fe_0.1_ NSs against 4T1 breast cancer cells. FITC‐labeled BOC‐Fe_0.1_ NSs were first incubated with 4T1 cells to evaluate the cellular uptake of the nanosheets. As demonstrated in Figure S15 (Supporting Information), BOC‐Fe_0.1_ NSs were effectively internalized by 4T1 cells within 4 h incubation. Subsequently, the in vitro anticancer effect of BOC‐Fe_0.1_ NSs was evaluated after 4 h incubation of cells with BOC‐Fe_0.1_ NSs. Standard methyl thiazolyl tetrazolium (MTT) cytotoxicity assay showed that the BOC‐Fe_0.1_ NSs itself has low cytotoxicity to 4T1 cells even at a high concentration (200 µg mL^−1^) (**Figure** **4**a). US alone (50 kHz; 1 W cm^−2^; 3 min) exhibited negligible damage to the 4T1 cells (Figure S16, Supporting Information), indicating that US alone did not induce mechanical injury of the cells. These results signify that BOC‐Fe_0.1_ NSs is biocompatible and can be internalised into cancer cells for intracellular SDT under US irradiation.

In vitro SDT antitumor performance was then evaluated for 4T1 cells incubated with BOC‐Fe_0.1_ NSs and exposed to different US powers (1, 2, and 3 W cm^−2^) for the irradiation time of 1, 2, and 3 min). As illustrated in Figure 4b, the viability of the cells decreased obviously with the increase of US irradiation time from 1 to 3 min, while the increase of US intensity further inhibited the cell growth. These results clearly showed that the tumor killing ability of BOC‐Fe_0.1_ NSs was US intensity and irradiation time dependent. Moreover, US irradiation at 1 W cm^−2^ for 3 min killed more than 50% cells. Therefore, 1 W cm^−2^ US irradiation for 3 min was selected for the following in vitro SDT antitumor performance tests. The antitumor performance of SDT was retained in an in vitro deep‐seated tumor model, where a piece of pork meat (∼1 cm thickness) was placed between the US probe and 4T1 cells (Figure 4c). The cell viability decreased remarkably in both groups (with/without pork tissue barrier), where the 4T1 cells were incubated with the increasing concentration of BOC‐Fe_0.1_ NSs and then treated with 1 W cm^−2^ US irradiation for 3 min. No significance of cell viability between the two groups was observed (Figure 4d), demonstrating that the US could efficiently penetrate through 1 cm of tissue and activate the BOC‐Fe_0.1_ NSs to generate high sono‐toxicity against 4T1 cells. To further demonstrate the deeper tissue penetration ability of SDT, a piece pork tissue (≈5.5 cm) was applied in the middle of cell culture dish and US probe (Figure S17a, Supporting Information). Greater than 50% of 4T1 cells were killed at the concentration of BOC‐Fe_0.1_ NSs higher than 50 µg mL^−1^ for both groups with and without pork tissue (Figure S17b, Supporting Information). Moreover, similar cell killing ability was observed for both groups, indicating that US‐based SDT could efficiently kill the cancer cells in deep‐seated tissue of more than 5 cm depth.

To investigate the intracellular ROS generation after various treatments, confocal fluorescence imaging of 4T1 cells stained with 2’‐7’‐dichlorofluorescin diacetate (DCFH‐DA) was obtained. As shown in Figure 4e, strong green fluorescence was observed in cells treated with BOC‐Fe_0.1_ NSs with H_2_O_2_, BOC‐Fe_0.1_ NSs with US, and BOC‐Fe_0.1_ NSs with H_2_O_2_ and US. In sharp contrast, intracellular green fluorescence of 4T1 cells treated with US only, H_2_O_2_ only, BOC‐Fe_0.1_ NSs alone, or H_2_O_2_ with US was not observed. The green signal in BOC‐Fe_0.1_ NSs + H_2_O_2_, BOC‐Fe_0.1_ NSs + US, and BOC‐Fe_0.1_ NSs + H_2_O_2_ + US groups confirmed the ROS generation by CDT, SDT, and CDT + SDT, respectively. In agreement with MTT data, the confocal images of 4T1 cells stained with fluorescein diacetate (FDA) and propidium iodide (PI) were recorded to determine the cell killing ability (Figure 4f). The cells’ treatment with BOC‐Fe_0.1_ NSs or BOC‐Fe_0.1_ NSs with H_2_O_2_ showed significant higher killing efficiency than other groups under same irradiation conditions. Altogether, these observations reveal that in this BOC‐Fe_0.1_ NS sonosensitiser system, 1) BOC‐Fe_0.1_ NSs is biocompatible with limited cytotoxicity even at high concentration (200 µg mL^−1^); 2) the US irradiation does not damage the normal cells while has deep penetration capability; 3) the ROS released by CDT and SDT has excellent cell killing ability.

**Figure 5:**
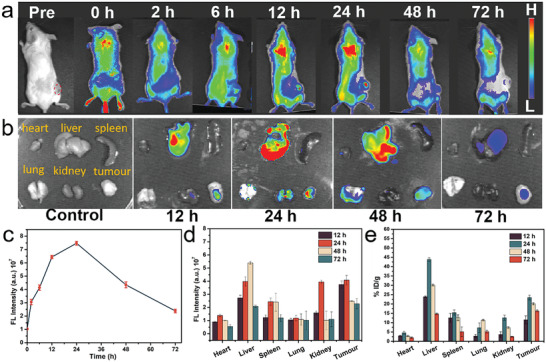
In vivo and ex vivo biodistribution analysis of BOC‐Fe_0.1_ NSs post the i.v. injection. a) In vivo fluorescence imaging of 4T1 tumor bearing mice at different time points after injecting Cy5.5 labeled BOC‐Fe_0.1_ NSs (dose: 10 mg kg^−1^). b) Ex vivo fluorescence images of heart, liver, spleen, lung, kidney, and tumor at different time points. c) Quantitative analysis of fluorescence intensity based on images in (a). d) Quantitative fluorescence intensity analysis of main organs and tumor based on images in (b) at 12, 24, 48, and 72 h. e) The ICP‐OES result of the Bi level in heat, liver, spleen, lung, kidney, and tumor collected at different time points from mice injected with BOC‐Fe_0.1_ NSs.

Prior to the in vivo tumor SDT evaluation, we investigated the accumulation of BOC‐Fe_0.1_ NSs at the tumor site by fluorescence imaging. After intravenous (i.v.) injection of Cy5.5 labeled BOC‐Fe_0.1_ NSs, 4T1 tumor bearing mice were subjected to fluorescence imaging at different time points (**Figure** **5**a). The changes of fluorescence signal at the tumor site showed that the tumor accumulation of BOC‐Fe_0.1_ NSs was time‐dependent (Figure 5b). The highest fluorescence intensity was observed at the 24 h, and then the fluorescence intensity decreased over the time (Figure 5c). These observations indicate that BOC‐Fe_0.1_ NSs achieved the most tumor accumulation at 24 h post the i.v. injection. As the time reached 72 h, the fluorescence intensity decreased substantially, suggesting metabolite decomposition or discharge via circulation in vivo. Accumulation of the BOC‐Fe_0.1_ NSs in the main organs and tumors were also examined by ex vivo fluorescence imaging of harvested tissues at 12, 24, 48, and 72 h post injection (Figure 5b,d). The tumor harvested at 24 h showed a relatively high fluorescence intensity, which is consistent with the fluorescence intensity from the in vivo imaging and inductively coupled plasma optical emission spectrometry (ICP‐OES) analysis of Bi (Figure 5e). Therefore, the optimal time window of US for SDT was determined to be 12–24 h post i.v. injection of BOC‐Fe_0.1_ NSs.

**Figure 6:**
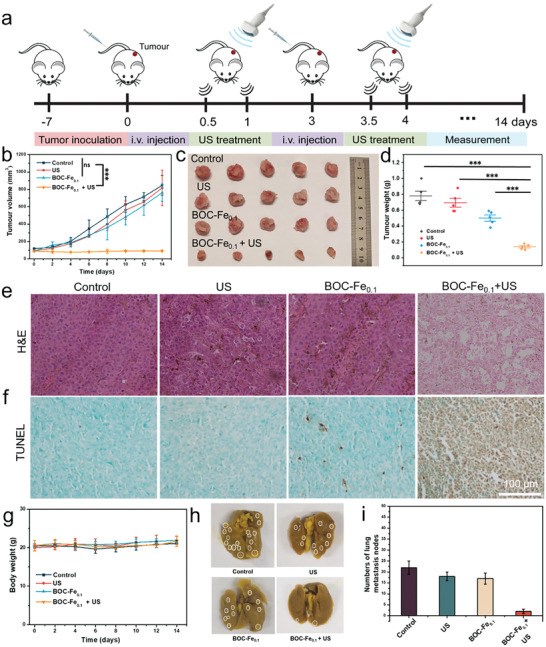
In vivo SDT against 4T1 breast cancer. a) In vivo treatment schedule of SDT after i.v. injection of BOC‐Fe_0.1_ NSs. b) Relative tumor growth curves based on the tumor volume change profiles of different groups of mice. c) Photo of tumors and d) the corresponding average weights of tumors which were dissected from mice at14 d after different treatments. e,f) H&E and TUNEL staining of tumor tissue sections images from different groups. g) Bodyweight of mice from different groups during various treatments. h) Photo of lungs dissected from mice of different groups. i) Number of lung nodes based on images (h).

Based on the in vivo and ex vivo biodistribution data, in vivo anti‐tumor treatment was further demonstrated using BOC‐Fe_0.1_ NSs as the sonosensitizer. The experimental procedures are depicted in **Figure** **6**a. Specifically, a breast cancer model was made by subcutaneously injecting 4T1 cells to the right back of female BALB/C mice. The tumor‐inoculated mice with the tumor volumes of ≈100 mm^3^ were randomly divided into four groups, including control (PBS), BOC‐Fe_0.1_ NSs, US, and BOC‐Fe_0.1_ NSs + US. The mice were i.v. injected with BOC‐Fe_0.1_ NSs at a dose of 10 mg kg^−1^. The tumor‐bearing mice were treated with US (50 kHz; 1 W cm^−2^; 3 min) at 12 h and 24 h after each injection of sonosensitizers. The tumor volume and body weight were measured every two days.

Figure 6b summarize the change of the tumor volume in these four groups of mice with different treatments. The tumor growth of BOC‐Fe_0.1_ NSs injected with US irradiation was significantly suppressed, i.e., the tumor volume was kept being constantly less than 100 cm^3^ in 14 days. The tumor growth in BOC‐Fe_0.1_ NSs only group also inhibited to a low degree due to the CDT effect. In sharp contrast, the tumor volume in control groups (PBS and US) reached ≈800–1000 cm^3^ at day 14 post treatment. The inhibition rate of BOC‐Fe_0.1_ NSs + US group was as high as 90% in comparison with the control group. The residual tumors collected at 14 days (Figure 6c,d) demonstrated the inhibited tumor growth by BOC‐Fe_0.1_ NSs alone, implying the CDT performance. The BOC‐Fe_0.1_ NSs + US treatment group showed the significant suppression of the tumor growth (Figure 6c) and the reduction of tumor weight (Figure 6c,d). Haematoxylin‐eosin (H&E) staining, and TdT‐mediated dUTP nick‐end labelling (TUNEL) assay of tumor sections showed that the BOC‐Fe_0.1_ NSs + US treatment induced a high level of cell apoptosis and/or necrosis in tumor tissues (Figure 6e,f), further verifying the therapeutic efficacy of SDT and CDT by BOC‐Fe_0.1_ NSs. Moreover, the body weight of mice had no noticeable change in all treatment groups (Figure 6g), indicating the excellent biocompatibility of BOC‐Fe_0.1_ NSs and high biosafety of the SDT/CDT. The H&E staining of the main organs of mice, including heart, liver, spleen, kidney, and lung, showed no additional obvious abnormalities and lesions in all treatment groups (Figure S18, Supporting Information), corroborating that the BOC‐Fe_0.1_ NSs can be used as a safe sonosensitiser for enhanced tumor therapy.

It is reported that 4T1 cells can be spontaneously transferred to other organs such as the lungs after inoculation in mice.^[^ ^33^ ^]^ Therefore, to evaluate the levels of lung metastasis during the tumor growth and treatment, lungs in different treatment group were collected and fixed by 4% paraformaldehyde containing 10% picric acid. The white nodule spots, i.e., metastasised tumors were labeled (Figure 6h). The quantitative data (Figure 6i) clearly showed that the number of nodules in the PBS control group (22.5 ± 3.1) is ten times that in the BOC‐Fe_0.1_ NSs + US treated group (2.2 ± 1.2). These observations confirmed that the SDT/CDT treatment efficiently suppressed the tumor lung metastasis.

## 3 Conclusion

In summary, a series of iron‐doped ultrathin BOC nanosheets with BSA coating on the surface were synthesized as the efficient SDT sonosensitizer for tumor treatment. In this nanosystem, the US activated the piezoelectric BOC NSs to produce intracellular ROS to induce tumor cell apoptosis and inhibit the tumor growth. The doped Fe^2+^/Fe^3+^ induced oxygen defects and reduced the bandgap, facilitating the ROS generation during US irradiation. Moreover, the Fe^2+^/Fe^3+^ doping enabled the nanosheets with catalytic activity to yield ·OH via Fenton reactions to further promote the cancer cell killing ability. More importantly, through regulating the amount of Fe doping, the oxygen defects on the BOC NSs were modulated, and the BOC‐Fe_0.1_ NS sonosensitizer was found to have the optimal charge transfer rate to enhance the SDT performance. The BOC‐Fe_0.1_ NS sonosensitizer showed excellent biocompatibility, while exhibited high ROS generation ratio to effectively kill the tumor cells within 3‐min US irradiation even with a 1 cm tissue barrier. With mild US irradiation, the BOC‐Fe_0.1_ NS sonosensitizer efficiently inhibited in vivo tumor growth and lung metastasis. Therefore, this work has not only reported a new piezoelectric BOC based ultrathin nanosheet as a promising cancer SDT agent, but also proposed a new strategy to develop uniform ultrathin nanosheets with metal ions doping and oxygen defects for enhanced SDT of deeply seated cancers.

## Conflict of Interest

The authors declare no conflict of interest.

## Supporting information

Supporting Information

## Data Availability

The data that support the findings of this study are available from the corresponding author upon reasonable request.

## References

[1] a) X. Tan , J. Huang , Y. Wang , S. He , L. Jia , Y. Zhu , K. Pu , Y. Zhang , X. Yang , Angew. Chem., Int. Ed. 2021, 60, 14051; 10.1002/anie.202102703 33797161

[2] a) X. Lin , S. Liu , X. Zhang , R. Zhu , S. Chen , X. Chen , J. Song , H. Yang , Angew. Chem., Int. Ed. 2020, 59, 1682; 10.1002/anie.201912768 31710768

[3] Z. Gong , Z. Dai , Adv. Sci. 2021, 8, 2002178. 10.1002/advs.202002178 PMC8132157 34026428

[4] W. Um , P. K. E. K , J. Lee , C. H. Kim , D. G. You , J. H. Park , Chem. Commun. 2021, 57, 2854. 10.1039/d0cc07750j 33625416

[5] N. Yumita , Y. Iwase , K. Nishi , H. Komatsu , K. Takeda , K. Onodera , T. Fukai , T. Ikeda , S.‐i. Umemura , K. Okudaira , Y. Momose , Theranostics 2012, 2, 880. 23082100 10.7150/thno.3899 PMC3475214

[6] X. Qian , Y. Zheng , Y. Chen , Adv. Mater. 2016, 28, 8097. 27384408 10.1002/adma.201602012

[7] a) S. Liang , X. Deng , G. Xu , X. Xiao , M. Wang , X. Guo , P. a. Ma , Z. Cheng , D. Zhang , J. Lin , Adv. Funct. Mater. 2020, 30, 1908598;

[8] J. Ouyang , L. Deng , W. Chen , J. Sheng , Z. Liu , L. Wang , Y.‐N. Liu , Chem. Commun. 2018, 54, 2874. 10.1039/c8cc00392k 29493688

[9] a) Y. Dong , S. Dong , B. Liu , C. Yu , J. Liu , D. Yang , P. Yang , J. Lin , Adv. Mater. 2021, 33, 2106838; 10.1002/adma.202106838 34655115

[10] X. Han , J. Huang , X. Jing , D. Yang , H. Lin , Z. Wang , P. Li , Y. Chen , ACS Nano 2018, 12, 4545. 29697960 10.1021/acsnano.8b00899

[11] a) Y. Liu , Y. Wang , W. Zhen , Y. Wang , S. Zhang , Y. Zhao , S. Song , Z. Wu , H. Zhang , Biomaterials 2020, 251, 120075; 32388168 10.1016/j.biomaterials.2020.120075

[12] a) S. Bai , N. Yang , X. Wang , F. Gong , Z. Dong , Y. Gong , Z. Liu , L. Cheng , ACS Nano 2020, 14, 15119; 33185089 10.1021/acsnano.0c05235

[13] a) A. A. Doinikov , A. Bouakaz , J Acoust Soc Am 2010, 127, 703; 20136192 10.1121/1.3279793

[14] Y. Zhao , T. Huang , X. Zhang , Y. Cui , L. Zhang , L. Li , Z. L. Wang , BMEMat 2023, 1, e12006.

[15] A.‐H. Liao , W.‐C. Ma , C.‐H. Wang , M.‐K. Yeh , Drug Deliv. 2016, 23, 2173. 25148541 10.3109/10717544.2014.951102

[16] D. F. Gaitan , L. A. Crum , C. C. Church , R. A. Roy , J Acoust Soc Am 1992, 91, 3166. 10.1121/1.402638 1556312

[17] E. A. Neppiras , B. E. Noltingk , Proc. Phys. Soc. Section B 1951, 64, 1032.

[18] K. Wang , C. Han , J. Li , J. Qiu , J. Sunarso , S. Liu , Angew. Chem., Int. Ed. 2022, 61, e202110429. 10.1002/anie.202110429 34612568

[19] X. Zhou , F. Yan , S. Wu , B. Shen , H. Zeng , J. Zhai , Small 2020, 16, 2001573. 10.1002/smll.202001573 32431007

[20] E. Lin , J. Wu , N. Qin , B. Yuan , Z. Kang , D. Bao , Catal. Sci. Technol. 2019, 9, 6863.

[21] X. Wang , J. Zhou , J. Song , J. Liu , N. Xu , Z. L. Wang , Nano Lett. 2006, 6, 2768. 17163703 10.1021/nl061802g

[22] R. Yu , L. Dong , C. Pan , S. Niu , H. Liu , W. Liu , S. Chua , D. Chi , Z. L. Wang , Adv. Mater. 2012, 24, 3532. 22544827 10.1002/adma.201201020

[23] M. Wu , Z. Zhang , Z. Liu , J. Zhang , Y. Zhang , Y. Ding , T. Huang , D. Xiang , Z. Wang , Y. Dai , X. Wan , S. Wang , H. Qian , Q. Sun , L. Li , Nano Today 2021, 37, 101104.

[24] B. Zhang , J. Zhang , R. Duan , Q. Wan , X. Tan , Z. Su , B. Han , L. Zheng , G. Mo , Nano Energy 2020, 78, 105340.

[25] a) M. Guan , C. Xiao , J. Zhang , S. Fan , R. An , Q. Cheng , J. Xie , M. Zhou , B. Ye , Y. Xie , J. Am. Chem. Soc. 2013, 135, 10411; 23782301 10.1021/ja402956f

[26] a) H. Zhang , J. Li , Y. Chen , J. Wu , K. Wang , L. Chen , Y. Wang , X. Jiang , Y. Liu , Y. Wu , D. Jin , W. Bu , Adv. Mater. 2021, 33, 2100472; 10.1002/adma.202100472 33759262

[27] Z. Zhong , C. Liu , Y. Xu , W. Si , W. Wang , L. Zhong , Y. Zhao , X. Dong , Adv. Healthcare Mater. 2022, 11, 2102632. 10.1002/adhm.202102632 35107866

[28] H. Li , J. Li , Z. Ai , F. Jia , L. Zhang , Angew. Chem., Int. Ed. 2018, 57, 122. 10.1002/anie.201705628 28635079

[29] Z. Wang , X. Mao , P. Chen , M. Xiao , S. A. Monny , S. Wang , M. Konarova , A. Du , L. Wang , Angew. Chem., Int. Ed. 2019, 58, 1030. 10.1002/anie.201810583 30417505

[30] a) X. Wang , X. Zhong , F. Gong , Y. Chao , L. Cheng , Mater. Horiz. 2020, 7, 2028;

[31] S. Mukasa , S. Nomura , H. Toyota , Jpn. J. Appl. Phys. 2004, 43, 2833.

[32] E. B. Flint , K. S. Suslick , Science 1991, 253, 1397. 17793480 10.1126/science.253.5026.1397

[33] H. Cao , Z. Dan , X. He , Z. Zhang , H. Yu , Q. Yin , Y. Li , ACS Nano 2016, 10, 7738. 27454827 10.1021/acsnano.6b03148

