# Supplementary material for: 2D Ultrathin Iron Doped Bismuth Oxychloride Nanosheets with Rich Oxygen Vacancies for Enhanced Sonodynamic Therapy
Source: Adv Healthc Mater. 2023 Jun 15;12(30):2301497. doi: 10.1002/adhm.202301497 (PMC11468327; doi:10.1002/adhm.202301497)
Supplement: Supplementary file 1 — Supporting Information [file ADHM-12-2301497-s001.pdf]

ADVANCED HEALTHCARE MATERIALS

Supporting Information

for *Adv. Healthcare Mater.*, DOI 10.1002/adhm.202301497

2D Ultrathin Iron Doped Bismuth Oxychloride Nanosheets with Rich Oxygen Vacancies for Enhanced Sonodynamic Therapy

Miaomiao Wu, Jiaxi Yong, Huayue Zhang, Zhiliang Wang, Zhi Ping Xu and Run Zhang**

Supporting Information

Two-dimensional ultrathin iron doped bismuth oxychloride nanosheets with rich oxygen vacancies for enhanced sonodynamic therapy

Miaomiao Wu,[a] Jiaxi Yong,[a] Huayue Zhang,[a] Zhiliang Wang,[a] Zhi Ping Xu,*[a,b] Run Zhang*[a]

[a] M. Wu, Dr. J. Yong, H. Zhang, Dr. Z. Wang, Prof. Z. P. Xu, Dr. R. Zhang

Australian Institute for Bioengineering and Nanotechnology (AIBN)

The University of Queensland

St Lucia, QLD 4072, Australia

E-mail: r.zhang@uq.edu.au; gordonxu@uq.edu.au

[b] Prof. Z. P. Xu

Institute of Biomedical Health Technology and Engineering and Institute of Systems and Physical Biology

Shenzhen Bay Laboratory

Shenzhen, 518107, P. R. China

Table of Contents

Experimental Procedures **3**
Chemicals and Reagents 3
Characterisation 3
Preparation of ultrathin BiOCl nanosheets (NSs) 3
Preparation of hydrothermal BiOCl NSs (hBOC) 3
Preparation of ultrathin iron doped BOC NSs (BiOCl-Fe NSs) 3
Surface modification of BiOCl-Fe NSs 3
Synthesis of FITC-labelled BOC-Fe NSs 4
Synthesis of Cy5.5-labeled BOC-Fe NSs 4
Reactive oxygen species (ROS) generation by US activation 4
Cellular experiments 4
Animal experiments 4
Evaluation of biodistribution 4
Characterization of BiOCl-Fe NSs **6**
References **15**

Experimental Procedures

Chemicals and Reagents

Bismuth(III) chloride ($BiCl_3$), tris(acetylacetonato)iron(III) (Fe(acac)$_3$), $Bi(NO_3)_3{\cdot}5H_2O$, hydrogen peroxide(H_2O_2), oleylamine (OM), oleic acid (OA), 1-octadecene (ODE), 1,3-diphenylisobenzofuran (DPBF), 3,3',5,5'-tetramethylbenzidine (TMB), 2,2,6,6-tetramethylpoperidine (TEMP), bovine serum albumin (BSA), fluorescein isothiocyanate (FITC), 2,7-dichlorofluorescein diacetate (DCFHDA), 4',6-diamidino-2-phenylindole (DAPI), Cy5.5-NHS ester, Calcein-AM and propidium iodide (PI) were purchased from Sigma-Aldrich. All chemicals and reagents used in this work were analytical grade and used without any further purification.

Characterisation

Nanoparticle morphology was visualized with TEM, STEM and AFM. The crystal structure of nanomaterials was analysed by the X-ray diffraction (XRD) patterns. XPS (Kratos axis supra plus XPS) and FTIR spectra (Nicolet 5700 ATR-FTIR spectrometer) were used to characterise surface chemical state and bonds. The piezo-electric property of nanomaterials was measured with piezoresponse force microscopy (PFM) in Cypher PFM. Specifically, TEM images were acquired in a Hitachi HT-7700 transmission electron microscope (TEM, Japan) operated at 100 kV. High-resolution TEM (HRTEM) micrographs and scanning transmission electron microscope (STEM) images in dark field were obtained in a Hitachi HF5000 operated at 200 kV. The XRD patterns were obtained using a Bruker D8 Advance powder XRD with monochromatized Cu Kα radiation (λ=1.5418 Å) in the 2-theta ranging from 10° to 80°. XPS spectra were recorded in a Kratos Axis Ultra XPS. Atomic Force Microscopy (AFM) image and piezoresponse force microscopy (PFM) images were measured in Cypher AFM. UV-vis absorption spectra were measured in a Perkin-Elmer Lambda 35 UV-vis spectrophotometer to determine the level of dye probes. Fluorescence spectra were measured on SHIMADZU RF-5301 PC spectrometer with excitation and emission slits of 5 nm. EPR spectra was measured by Bruker Elexsys E500 to detect ·OH, $^1O_2$ and $\cdot O_2^-$. CLSM images were captured by a Leica SP8 confocal microscope. Flow cytometry assays were obtained by a CytoFLEX flow cytometer. In vivo and ex vivo fluorescence images were recorded by The IVIS Lumina X5 imaging system.

Preparation of ultrathin BiOCl nanosheets (NSs)

BiOCl NSs were synthesised by a robust one-pot thermal decomposition approach. Typically, under a nitrogen atmosphere, 0.1 g bismuth (III) chloride was dissolved in a mixed solvent of 6 mL oleylamine, 5 mL octadecene and 500 µL oleic acid under vigorous stirring at 100 °C for 30 min. The dark grey opaque solution was then heated to 160 °C with string for 20 min. After cooling down to room temperature, the dark grey product was collected by centrifugation at 9,000 rpm for 10 min. The as synthesised BiOCl NSs were washed five times with isopropanol and ethanol, followed by drying in vacuum at 60 °C overnight for further characterisation.

Preparation of hydrothermal BiOCl NSs (hBOC)

hBOC were synthesised according to solvothermal method described previously [1]. Typically, 0.486 g $Bi(NO_3)_3{\cdot}5H_2O$ and 0.400 g PVP were dissolved in 25 mL mannitol solution (0.1 M) with vigorous stirring for 10 min. Saturated NaCl solution (5 mL) was then added into the above mixture solution. After 10 min stirring, the mixture was transferred to a Teflon-lined stainless-steel autoclave of 45 mL and heated to 160 °C for another 3h. After cooling to the room temperature, the white solid product was collected by centrifugation (9,000 rpm, 5 min) and washed with deionized water three times. The final products were dried at 60 °C overnight for further characterization.

Preparation of ultrathin iron doped BOC NSs (BiOCl-Fe NSs)

The procedure for synthesis of BiOCl-Fe NSs is similar to that of above ultrathin BiOCl NSs. In the BiOCl-Fe NSs synthesis, iron acetylacetonate (0.025, 0.05, 0.1 and 0.2 g) was added into the mixture. Followed by the similar heating and stirring procedures of BOC NSs preparation, BOC-$Fe_{0.05}$, BOC-$Fe_{0.1}$, BOC-$Fe_{0.2}$, and BOC-$Fe_{0.4}$ NSs were obtained.

Surface modification of BiOCl-Fe NSs

To improve the biocompatibility of BiOCl-Fe NSs and colloidal stability, BSA coating was applied to modify the surface to obtain BiOCl-Fe@BSA NSs (BOC-Fe NSs). In a typical method, 1 mL of BiOCl-Fe NSs suspension (4 mg/mL) was dropwise added into 4 mL of BSA solution (10 mg/mL). The mixture was vigorously stirred at room temperature for another 3 h before centrifugation and washing with deionized water for five times. The final product was collected and re-dispersed in filtered PBS buffer for further use.

Synthesis of FITC-labelled BOC-Fe NSs

To label the BOC-Fe NSs with FITC, a mixture of 1 mg FITC in 5 mL ethanol was added dropwise into the BOC-Fe NSs (2 mg/mL) and the solution was stirred at room temperature for 2 h. The free FITC dye was then removed by centrifugation and the pellet was washed with deionized water for another three times. The obtained FITC-labelled BOC NSs was dispersed in PBS buffer and stored at 4 °C in dark for further utilize.

Synthesis of Cy5.5-labeled BOC-Fe NSs

To label the BOC-Fe NSs with Cy5.5, 1 mg Cy5.5 monofunctional N-hydroxysuccinimide ester (Cy5.5-NHS) was first dissolved in 1 mL phosphate buffer of pH 8.0. 30 μL of the above solution was then added into BOC-Fe NSs (1 mg/mL) and the mixture was stirred at room temperature for 24 h. The free Cy5.5 dye was removed by centrifugation and the pellet was washed with deionized water for three times. The resulting Cy5.5-conjugated BOC-Fe NSs were dispersed in PBS buffer and stored at 4 °C in dark for further use.

Reactive oxygen species (ROS) generation by US activation

Upon US activation, DPBF was used as a probe for detection of singlet oxygen ($^1O_2$) generation. Specifically, BOC/BOC-Fe NSs (30 μg/mL) and DPBF (20 μg/mL) were dispersed in 2 mL of PBS (pH = 7.4). After different US durations (50 kHz; 1 W cm^{-2}), the absorbance of DPBF at 416 nm was measured by UV-vis.

Similarly, DHE fluorescence probe was used for detection of $\bullet O_2^-$ generation by US activation. The mixture of BOC-Fe NSs (30 μg/mL) and DHE (20 μg/mL) in 2 mL of PBS (pH = 7.4) was applied with US (50 kHz; 1 W/cm^2). At different US durations, the fluorescence spectra of DHE were recorded by fluorimeter.

For detection of •OH, BOC-Fe NSs (30 μg/mL) and TMB (20 μg/mL) were dispersed in 2 mL of PBS (pH = 7.4). After different US durations (50 kHz; 1 W cm^{-2}), the absorbance of TMB at 652 nm was measured by UV-vis to evaluate the production rate of •OH.

Cellular experiments

The cytotoxicity of BOC-Fe NSs and corresponding relative cell viabilities post treatment were determined by the standardized MTT assay. For fluorescence imaging analysis of cellular internalisation of BOC-Fe NSs, breast cancer 4T1 cells (1×10^5 cells per well) were cultured overnight in a 6-well culture dish with a cover glass at the bottom. After that, 1 mL RPMI-1640 containing FITC-labelled BOC-$Fe_{0.1}$ NSs (50 μg/mL) was added into the culture dish and the cells were further incubated for another 2, 4, and 8 h. The cells were then fixed by mounting medium with DAPI before subjecting to fluorescence imaging by a CLSM.

Upon the US activation, intracellular ROS detection was performed using DCFH-DA as the fluorescence probe. The cells were randomly divided into seven groups, including (G1) control, (G2) US, (G3) H_2O_2, (G4) H_2O_2 + US, (G5) BOC-Fe, (G6) BOC-Fe + US, (G7) BOC + H_2O_2 (50 μM) + US (50kHz; 1 W cm^{-2}; 3 min). The treatment of each group was followed by the staining with DCFH-DA for 20 min. After washing with PBS for three times, the cells in each group were subjected to fluorescence imaging by CLSM. To detect the cell death caused by US treatment, the cells were stained with calcein AM (green colour) and propidium iodide (red colour). The fluorescence images were acquired by a CLSM.

Animal experiments

To establish the in vivo tumour model, female BALB/c mice were subcutaneous injected with 4T1 cells (100 μL, 1×10^7 cells per mL). After the tumour volume reached 100 mm^3 (~7 days post injection of 4T1 cells) (set as day 0), the mice were randomly divided into four groups (*n* = 5 per group), including (G1) saline, (G2) US, (G3) BOC-$Fe_{0.1}$ (10 mg/kg), and (G4) BOC-$Fe_{0.1}$ (10 mg/kg) + US. For G4, BOC-$Fe_{0.1}$ dispersed in saline was intravenously injected into mice at day 0. The US (50 kHz; 1 W cm^{-2}; 3 min) was conducted after 12 and 24 h of BOC-$Fe_{0.1}$ NSs injection. Same US treatment was applied to G2 except the administration of BOC-$Fe_{0.1}$ NSs. Body weight and tumour volume of all mice were recorded every 2 days. The tumour volume was obtained using the equation, volume = length × $width^2$ × 0.5. After 2 weeks, all the mice were euthanized to collect the tumour and main organs (heart, liver, spleen, lungs, and kidneys), which were further sectioned into slices for H&E and TUNEL staining.

Evaluation of biodistribution

4T1 tumour-bearing mice (n = 8) were intravenously injected with Cy5.5-labeled BOC-$Fe_{0.1}$ (10 mg/kg). Those mice were imaged at 2, 4, 6, 12, 24, 48, and 72 h by the IVIS optical imaging system. At 12, 24, 48, and 72 h post-injection, the mice were euthanized. Subsequently, their main organs and tumour were collected for the ex vivo imaging. After that, the organs were weighted and digested in a mixed solution of HNO_3 and HCl. The biodistribution in organs and tumours were calculated as Bi percentage of the injected dose per gram of tissue.

Characterization of BiOCl-Fe NSs

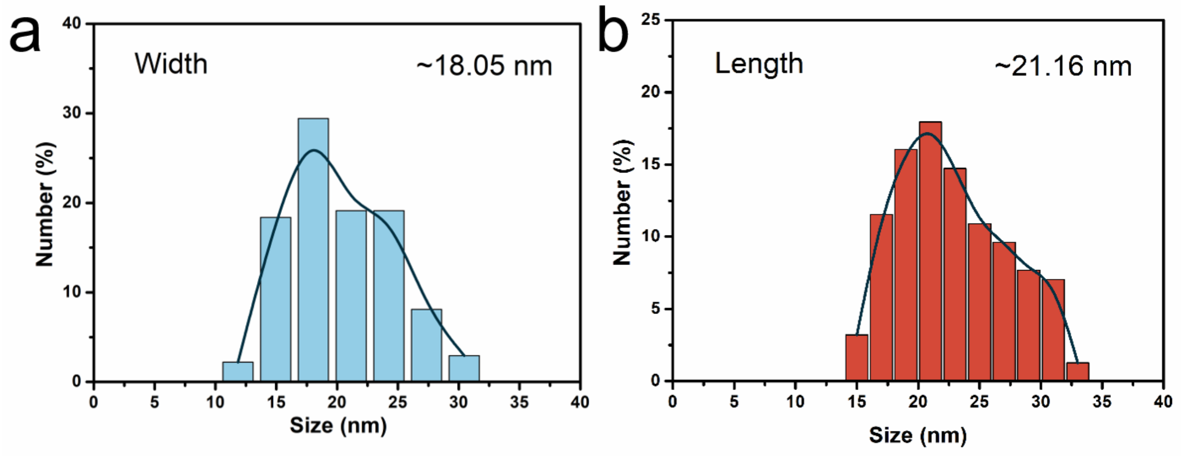

Figure S1. Width (a) and length (b) distributions of BiOCl-$Fe_{0.1}$ NSs determined by TEM images.

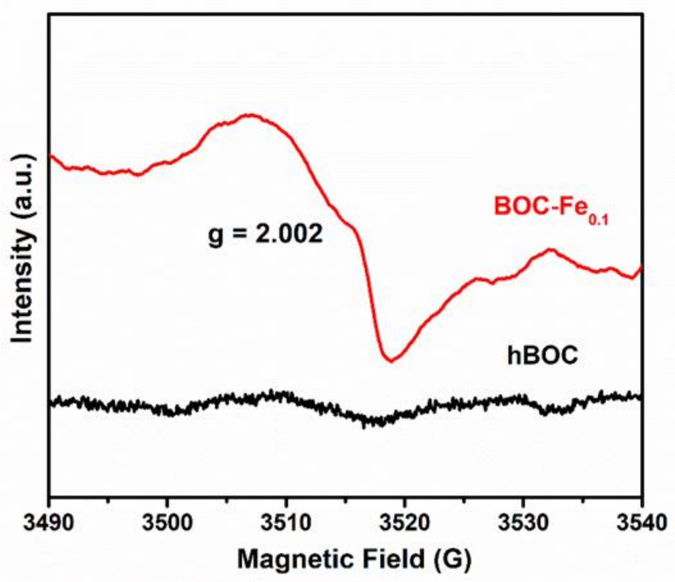

Figure S2. EPR analysis of BOC-$Fe_{0.1}$ and hBOC.

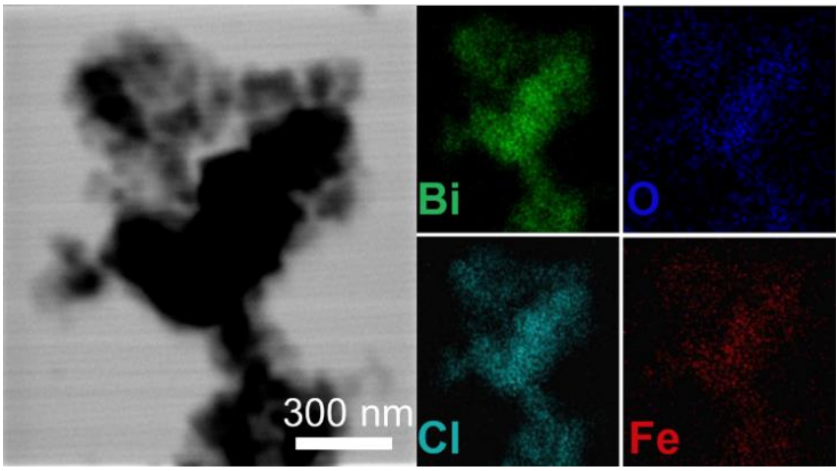

Figure S3. TEM image of specific area of of BiOCl-$Fe_{0.1}$ NSs and the corresponding elemental mapping images of BiOCl-$Fe_{0.1}$.

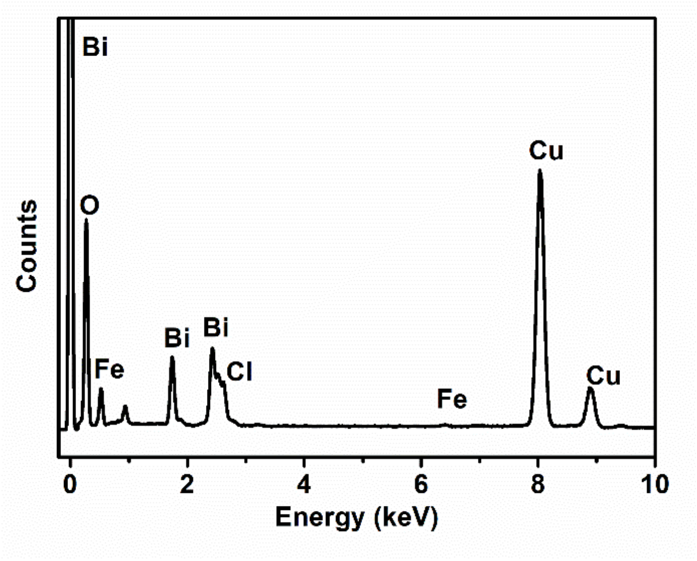

Figure S4. EDS spectrum of BiOCl-$Fe_{0.1}$ NSs on the copper foam.

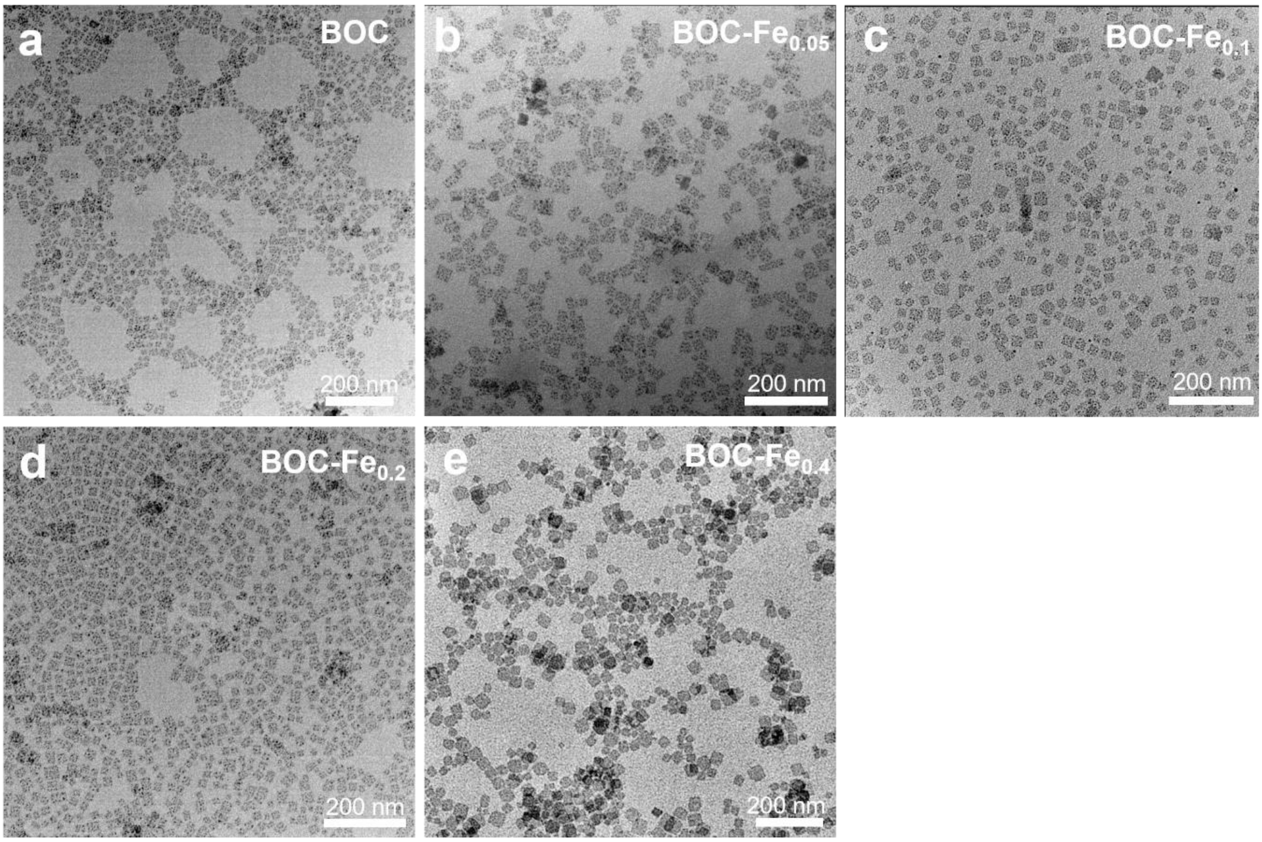

Figure S5. TEM images of pure BiOCl NSs (a), BiOCl-$Fe_{0.05}$ NSs (b), BiOCl-$Fe_{0.1}$ NSs (c), BiOCl-$Fe_{0.2}$ NSs (d), and BiOCl-$Fe_{0.4}$ NSs (e).

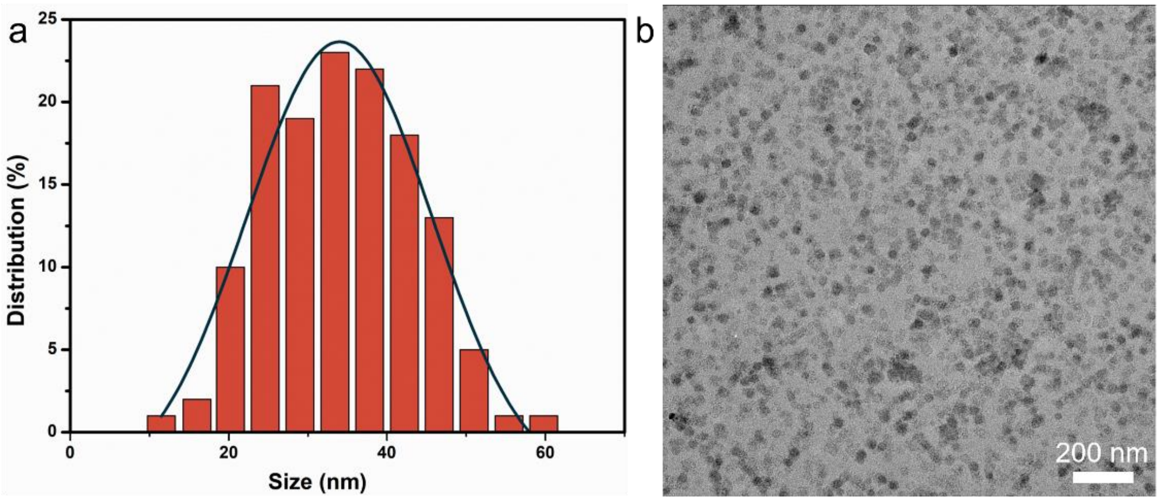

Figure S6. Size distribution (a) and TEM image (b) of BSA coated BiOCl-Fe NSs (BOC-Fe NSs).

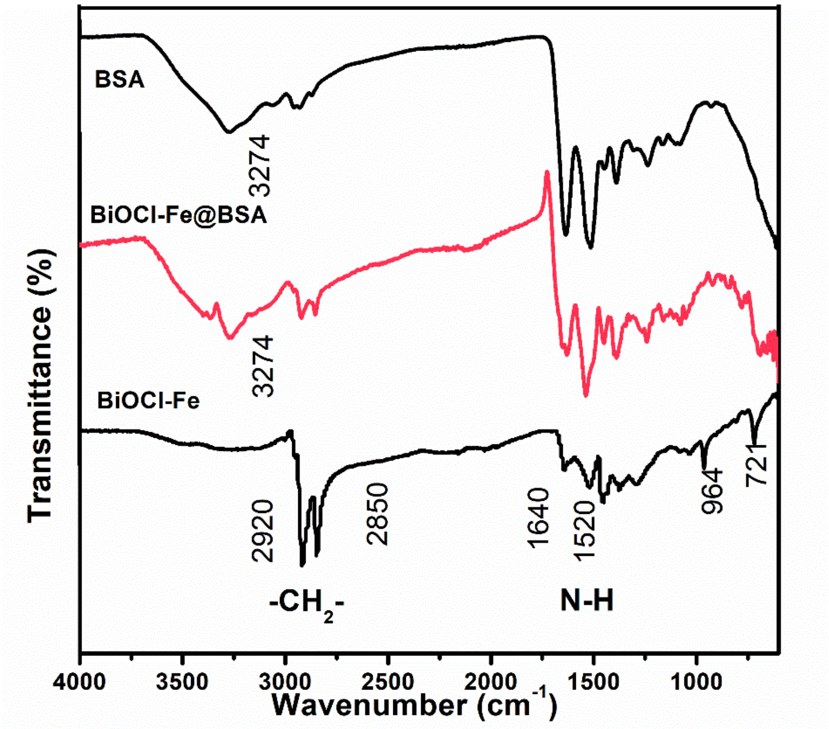

Figure S7. FTIR spectra of pure BSA (top), bare BiOCl-Fe NSs, (bottom) and BiOCl-Fe@BSA (BOC-Fe) (middle).

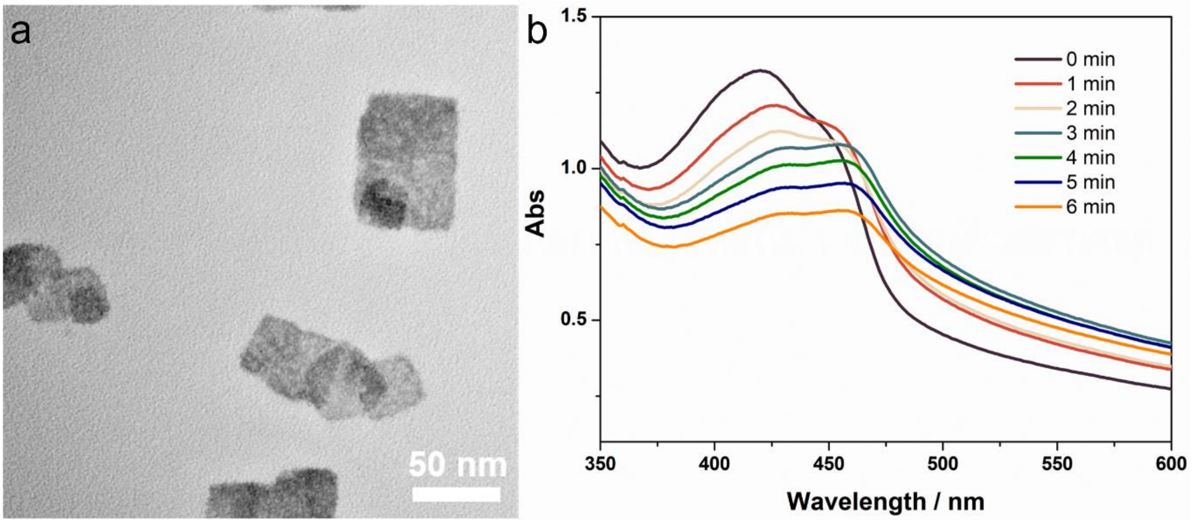

Figure S8. (a) TEM image of hydrothermal BiOCl NSs (hBOC) and (b) UV-Vis absorption spectra of DPBF and hBOC mixture under ultrasound at different times.

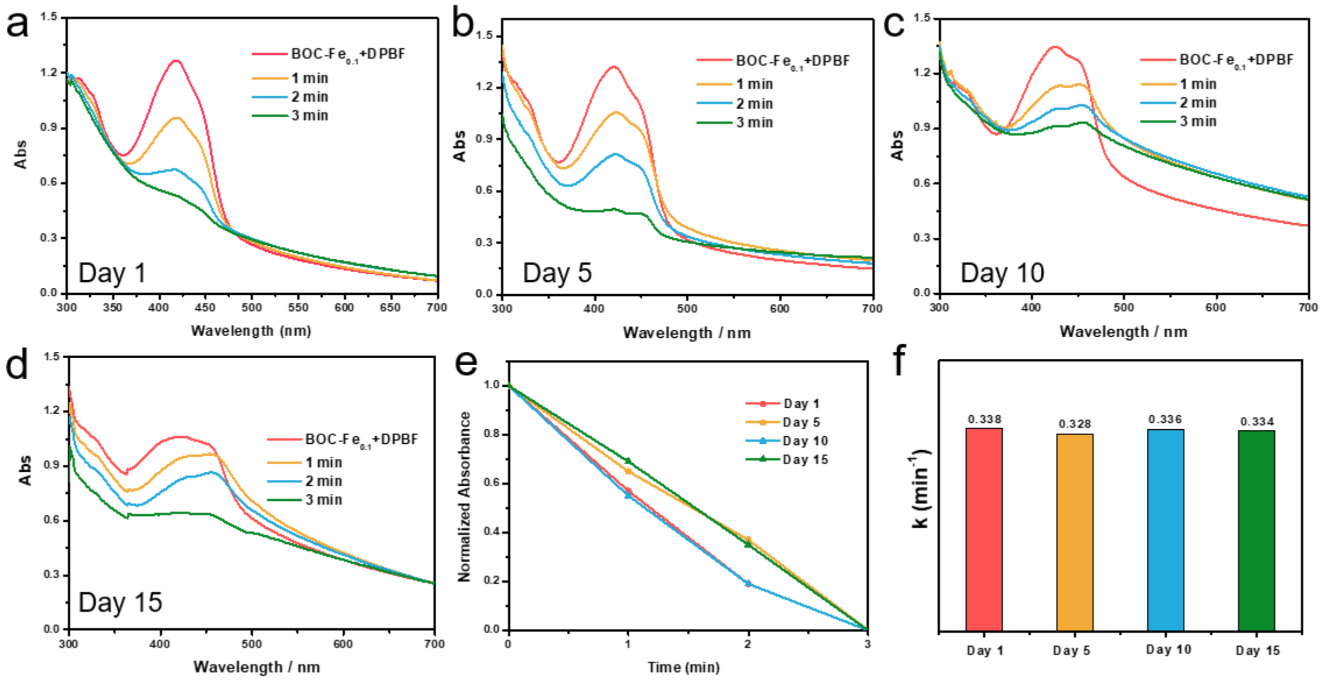

Figure S9. UV-Vis absorption spectra of DPBF and BOC-Fe mixture under ultrasound at different times (1 min, 2 min, 3min) every 5 days: day 1 (a), day 5 (b), day10 (c), day 15 (d), and the corresponding absorbance decreased over time (e). (f) The corresponding ROS production rate at different days.

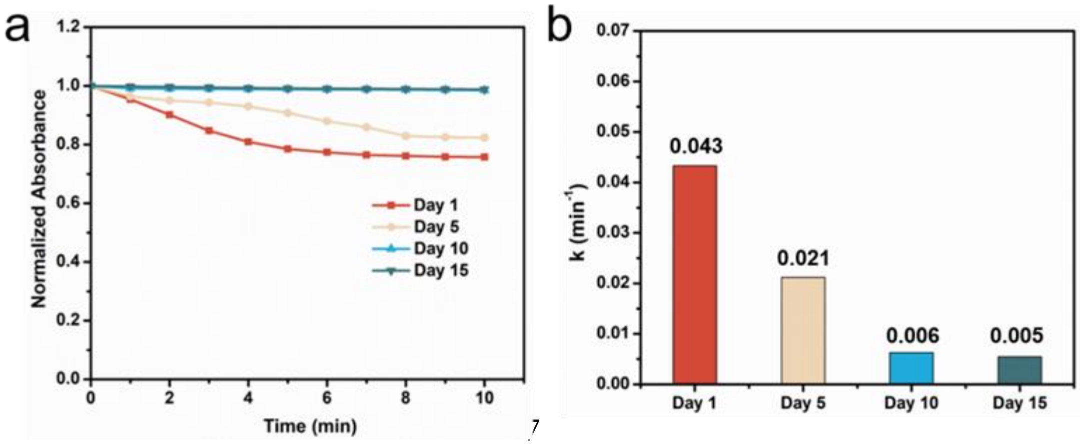

Figure S10. The decrease of DPBF's absorbance of RB solution under ultrasound irradiation at different time (1 min -10 min) every 5 days (a) and the corresponding ROS generation rate (b).

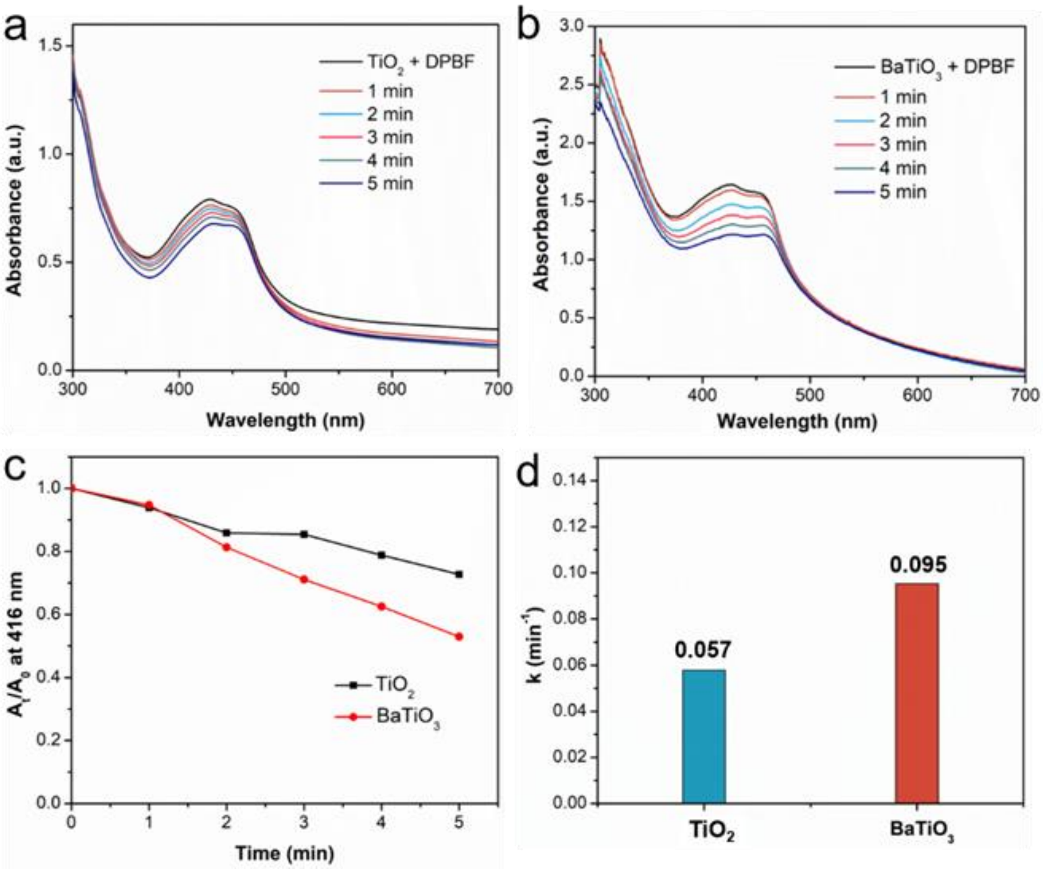

Figure S11. SDT performance of TiO_2 (a) and $BaTiO_3$ (b) measured by using the DPBF probe. (c,d) Comparison of SDT effects of TiO_2 and $BaTiO_3$.

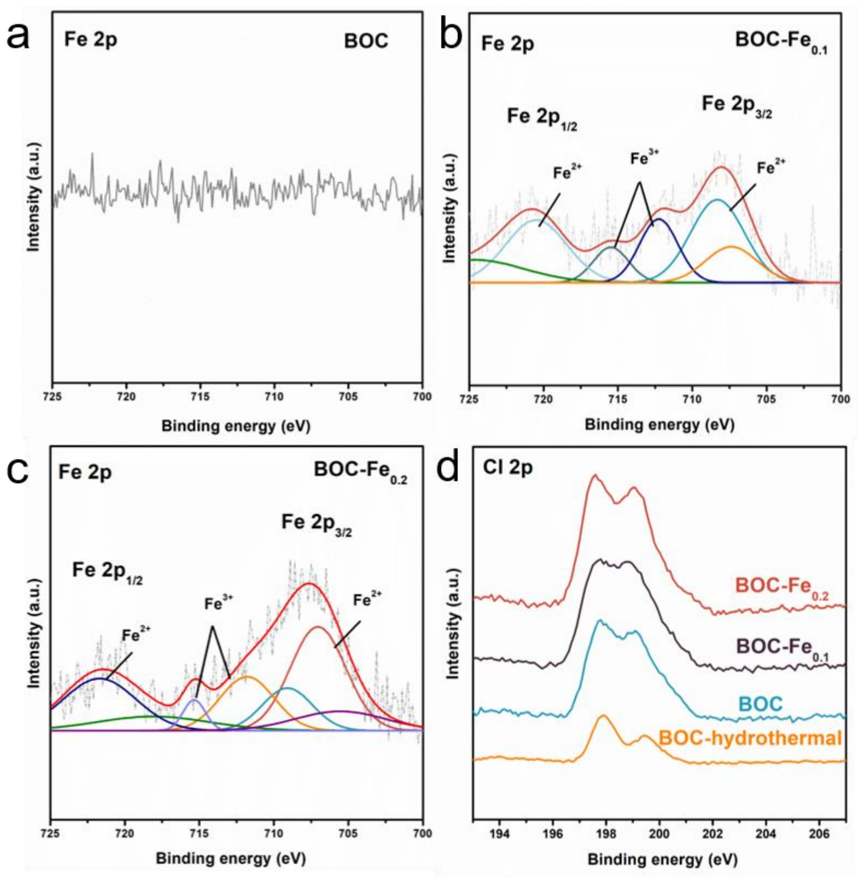

Figure S12. XPS analysis of BOC and BOC-Fe. XPS analysis of BOC (a) shows no Fe present. XPS analyses of BOC-$Fe_{0.1}$ (b) and BOC-$Fe_{0.2}$ (c) reveal that Fe^{2+} and Fe^{3+} exist in the BOC-Fe NSs, and (d) XPS analysis of element Cl for hBOC, BOC, and BOC-Fe.

Table S1. The specific ratio of lattice oxygen (O_L), oxygen vacancy (O_V), and surface absorbed oxygen species (O_A) analysed by XPS.

Sample	O_L	O_V	O_A
hBOC	76%	9%	15%
BOC	50%	30%	20%
BOC-$Fe_{0.1}$	42%	24%	34%
BOC-$Fe_{0.2}$	52%	19%	29%

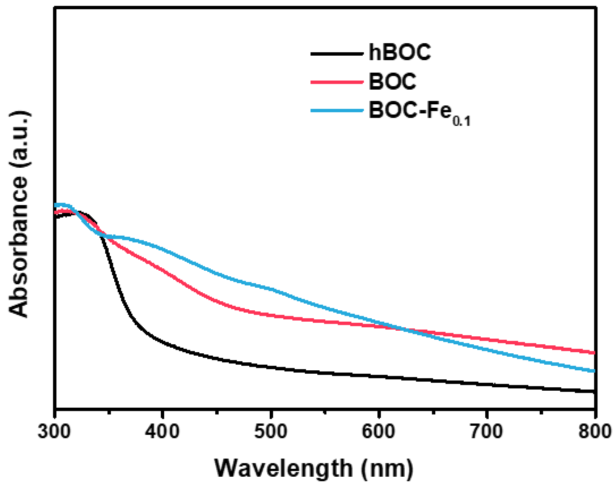

Figure S13. The solid ultraviolet spectra of BOC-Fe, BOC, and hBOC.

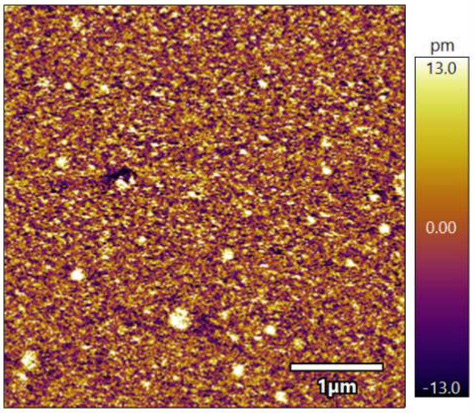

Figure S14. Amplitude image of BOC-Fe by PFM analysis.

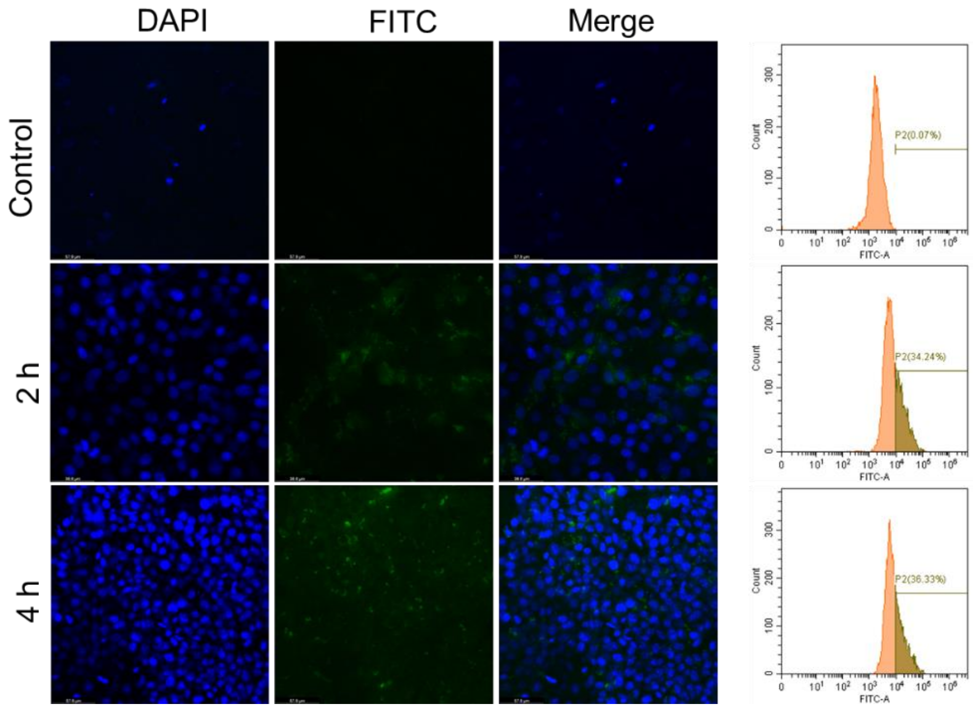

Figure S15. Cell (4T1 cells) uptake of FITC-labelled BOC-$Fe_{0.1}$ NSs at 2 and 4 h.

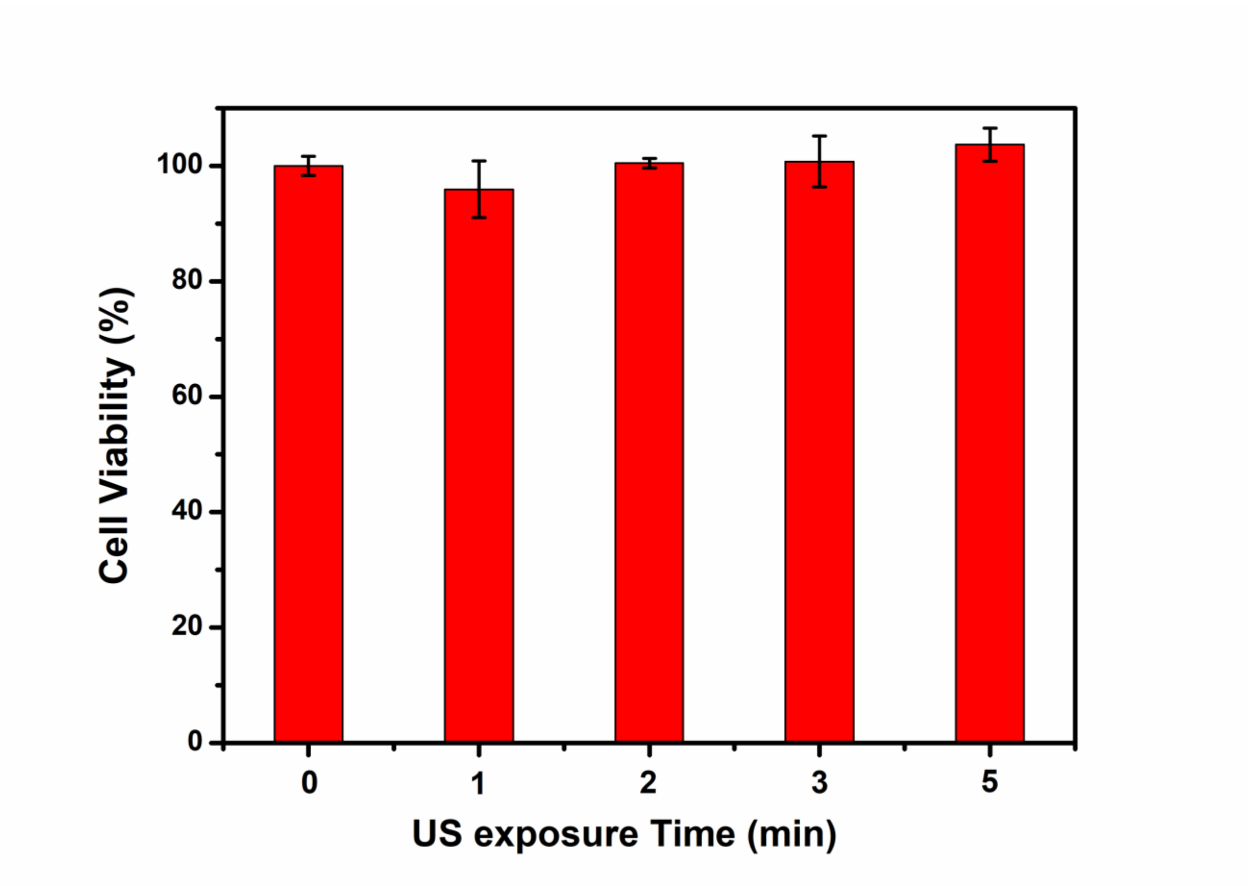

Figure S16. Relative viability of 4T1 cells after treated with US (50 kHz; 3 W cm^{-2}) from 0 – 5 min.

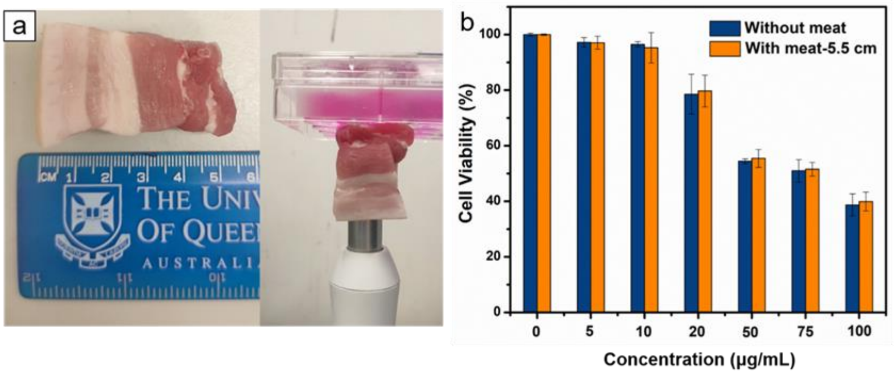

Figure S17. (a) Image of pork meat and in vitro simulated deep tissue model of SDT. (b) Relative viability of BOC NSs incubated 4T1 cells after SDT with and without meat (about 5.5 cm).

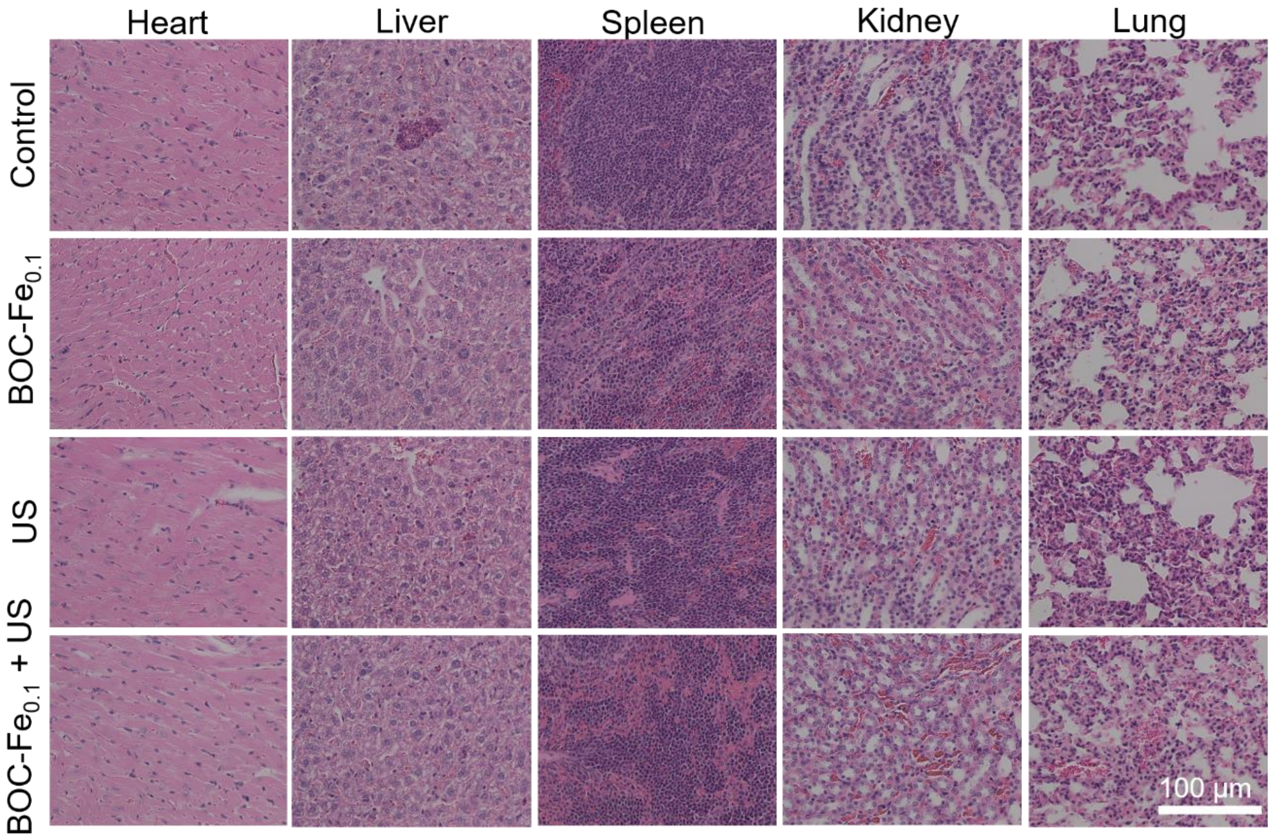

Figure S18. Representative images of H&E staining of tumour tissues in different treatments.

References

[1] M. Guan, C. Xiao, J. Zhang, S. Fan, R. An, Q. Cheng, J. Xie, M. Zhou, B. Ye, Y. Xie, *J. Am. Chem. Soc.* **2013**, *135*, 10411-10417.
